# Degludec: the new ultra-long insulin analogue

**DOI:** 10.1186/s13098-015-0037-0

**Published:** 2015-06-26

**Authors:** Marcos Antonio Tambascia, Freddy Goldberg Eliaschewitz

**Affiliations:** Faculty of Medical Sciences, State University of Campinas, São Paulo, Brazil; Hospital Israelita Albert Einstein – São Paulo Brazil, and CPClin Clinical Research Center, Rua Goias 91, São Paulo, CEP01244-030 Brazil

**Keywords:** Extended-action insulin analogues, Degludec, Hypoglycemia

## Abstract

The development of extended-action insulin analogues was motivated by the unfavorable pharmacokinetic (PK) profile of the conventional long-acting insulin formulations, generally associated with marked inter and intra patient variability and site- and dose-dependent effect variation. The new ultra-long insulin analogue degludec (IDeg) has the same amino acid sequence as human insulin except for the removal of threonine in the position 30 of the B chain (*Des*-B30, “De”) and the attachment, via a glutamic acid linker (“glu”), of a 16-carbon fatty diacid (hexadecanoic diacid, “dec”) to lysine in the position 29 of the B chain. These modifications allow that, after changing from the pharmaceutical formulation to the subcutaneous environment, IDeg precipitates in the subcutaneous tissue, forming a depot that undergoes a highly predictable gradual dissociation. Thus, once-daily dosing of IDeg results in a low peak: trough ratio, with consequent low intra-individual variability and plasmatic concentrations less critically dependent upon the time of injections. The clinical development program of IDeg (BEGIN) was comprised of 9 therapeutic confirmatory trials of longer duration (26–52 weeks) and showed that the efficacy of IDeg is comparable to insulin glargine in type 1 (T1D) and type 2 (T2D) diabetes patients across different age, body mass index and ethnic groups. This new ultra-long insulin analogue presents as advantages flexibility in dose timing and lower risk of hypoglycemia.

## Introduction

In spite of the body of evidence demonstrating the importance of tight glycemic control in the prevention of chronic complications of diabetes mellitus (DM), achieving the targets recommended by the Diabetes Societies’ Guidelines remains a difficult task. Among the reasons proposed to explain this clinical challenge are factors related to the disease, such as the progressive nature of DM, and factors related to the patients, such as lack of proper diabetes education and, consequently, poor adherence to therapy and self-management. Additionally, insulin administration by subcutaneous route has intrinsic limitations that, together with the pharmacokinetic (PK) profile of insulin formulations, do not reproduce the physiological patterns of insulin secretion [1].

Insulin therapy has greatly evolved in the last 40 years or so; starting with bovine and porcine impure insulin preparations, to those highly purified (monocomponent insulins) and to humanized porcine insulin, it was only in the late 70s, with the advent of the recombinant DNA technology, that insulin could be biosynthesized. Recombinant DNA technology paved the way for the synthesis of insulin analogues, molecules that contain rearrangements in amino acids position to modify the PK profile of insulin, better mimicking prandial (rapid-acting insulin analogues) and basal (extended-action insulin analogues) insulin secretion [2].

The first successful attempt to prolong the action of soluble short-term insulin was its non-covalent binding to protamine, decreasing its solubility at physiological pH and delaying its absorption from the subcutaneous tissue, which resulted on intermediate-acting NPH (neutral protamine Hagedorn) insulin. Addition of varying amounts of zinc salts without protamine also reduces the solubility of insulin and originated the Lente family of insulin [3]. The main drawbacks associated with these long-acting formulations are the substantial inter and intra patient variability, accounting for a large proportion of the day-to-day glycemic fluctuation [4]; their marked site- and dose-dependent effect variation; and the variability associated with the magnitude of resuspension before injection [5]. All these limitations, combined with the inherent ability of insulin therapy to cause hypoglycemia and weight gain, stimulated the development of the new insulin analogues, glargine and detemir. Glargine (IGlar) is a di-arginyl insulin analogue whose isoeletric point increased from 5.4 towards a neutral pH, with consequent precipitation at the site of injection, delayed absorption and prolonged effect. The mechanism of protraction of detemir involves deletion of Thr B30 and covalent acylation of Lys B29, that determines reversible binding of insulin to albumin, delayed absorption and prolonged effect [2].

### Structure and properties underlying the mechanism of insulin protraction in degludec formulation

During the synthesis process in pancreatic β-cells, insulin molecules self-associate into dimers that, in presence of zinc, assemble into hexamers (comprised of three dimers arranged around two zinc ions) allowing efficient storage into the secretory granules. After exocytosis, dilution immediately dissociates the hexamers in dimmers, and then in the biologically active monomers [6]. These properties of hexamers formation and dissociation are exploited for the pharmaceutical development of insulin analogues to accelerate or slow down the rate at which insulin leaves the subcutaneous injection site to bloodstream [7].

**Fig. 1:**
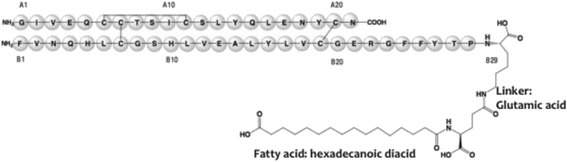
The structural formula of insulin degludec. Adapted from Reference [15]

**Fig. 2:**
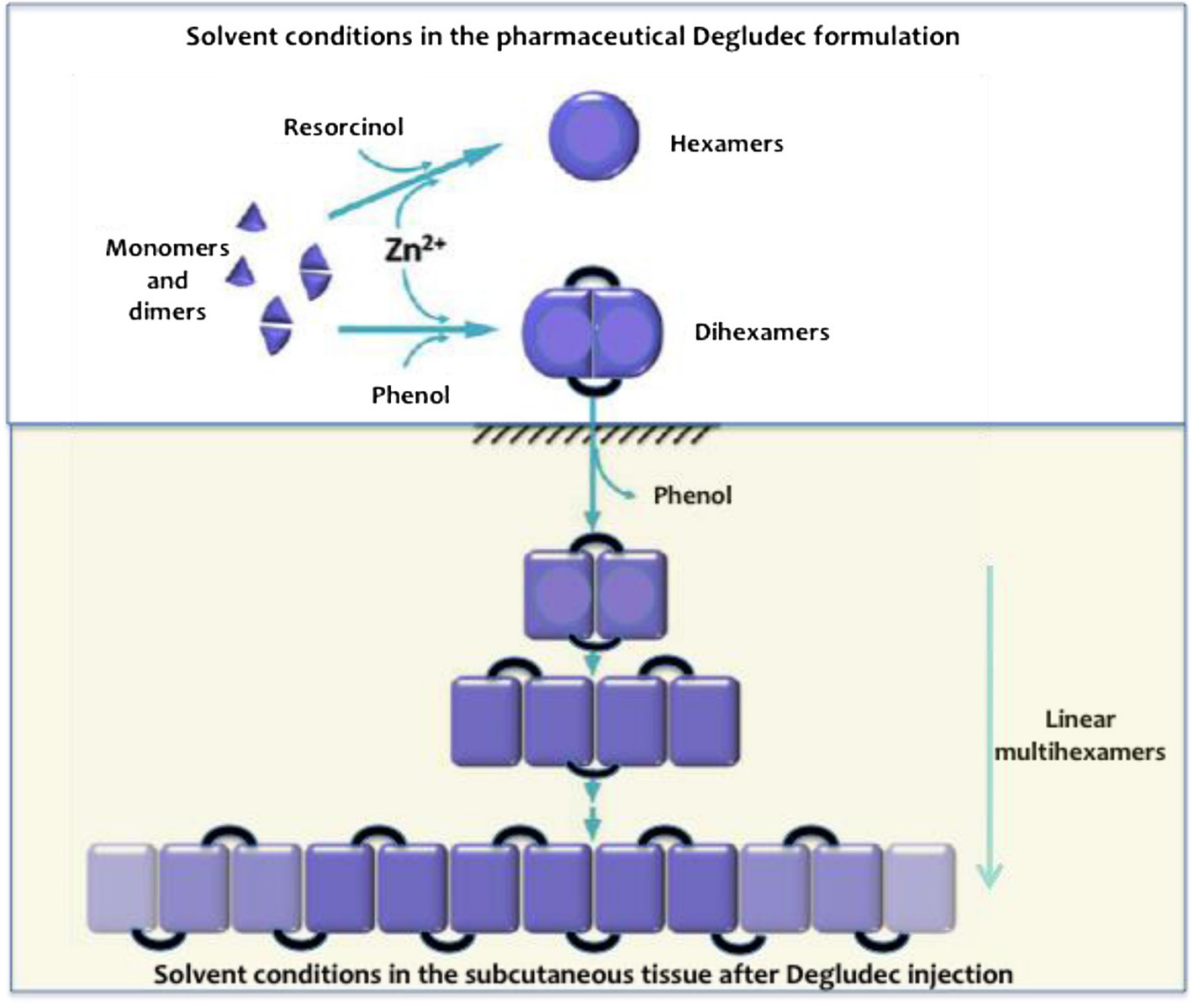
In the solvent conditions in insulin degludec formulation, the presence of zinc and resorcinol determines the formation of hexamers, and the presence of zinc and phenol determines the formation of dihexamer (upper panel). In the subcutaneous tissue after insulin degludec injection, phenol depletion promotes the self-association of dihexamers to form linear multihexamers, which will precipitate (lower panel). The black bars between degludec hexamers represent the acyl modification of LysB29. Adapted from Reference [8] (Steensgaard et al.)

The new ultra-long insulin analogue degludec (IDeg) has the same amino acid sequence as human insulin except for the removal of threonine in the position 30 of the B chain (*Des*-B30, “De”) and the attachment, via a glutamic acid linker (“glu”), of a 16-carbon fatty diacid (hexadecanoic diacid, “dec”) to lysine in the position 29 of the B chain (Fig. 1). In IDeg formulation, the presence of zinc and resorcinol determines the formation of hexamers, and the presence of zinc and phenol determines the formation of dihexamers. After IDeg injection, phenol depletion promotes the self-association of dihexamers to form linear multihexamers, which precipitate in the subcutaneous tissue [8] (Fig. 2). The acylation of lysine B29 also participates in the protraction mechanism, permitting binding to albumin in the bloodstream, like insulin detemir [9]. Slow dispersion of zinc ions from the subcutaneous depot allows a highly predictable gradual dissociation into insulin monomers, which behave exactly like human insulin regarding insulin receptor binding and activation and the subsequent metabolic effects. Following IDeg injection, insulin concentrations rise immediately but slightly, achieving maximum plasmatic concentration (C_max_) after 10–12 h; the mean terminal half-life (t_1/2_) of 17–25 h is almost twice as that of IGlar, until now the longest-acting insulin analogue (Fig. 1) [10].

IDeg concentrations achieve steady state after 2–3 days of once-daily administration with no further accumulation thereafter because, from that point, the daily-injected dose equals the daily-eliminated amount of insulin when repeated equivalent doses are administered at adequate intervals [11].

**Fig. 3:**
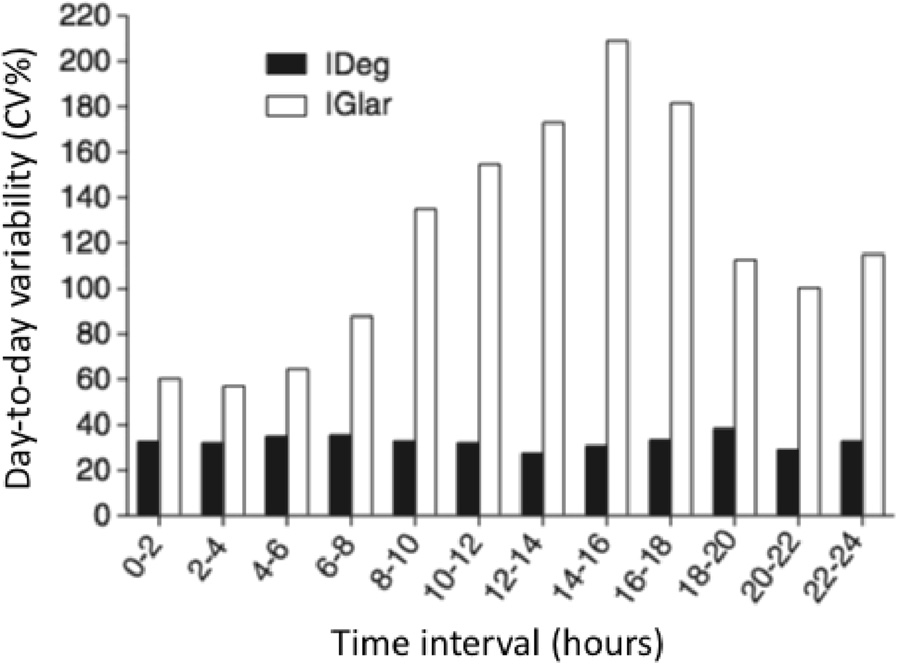
Day-to-day variability in the glucose - lowering effect of insulin degludec (IDeg) and insulin glargine (IGlar) over 24 h at steady state as shown by the coefficient of variation (CV) for the pharmacodynamic endpoint (area under the curve for glucose infusion rates from 0–24 h). Adapted from Reference [13] (Heise et al.)

Concerning intra-individual variability, the insulin analogues IGlar and detemir already present lower within-subject variability than NPH, as demonstrated by Heise et al. in type 1 diabetes (T1D) patients distributed in three groups for receiving each one of these long-acting formulations (0.4 U/kg once daily on four identical study days) under euglycemic clamp conditions. The coefficient of variation (CV) for the pharmacodynamic endpoint GIR-AUC _(0-24h)_ (area under the curve for glucose infusion rates from 0–24 h) was 68 % for NPH, 48 % for IGlar and 27 % for detemir [12]. Heise et al. [13] also compared intra-individual variability between IDeg and IGlar in T1D patients, now under steady-state conditions (which might explain the different results observed for IGlar), in 24-h euglycemic clamps performed on the 6^th^, 9^th^ and 12^th^ day of treatment with 0.4 U/kg of each one of the insulin preparations. The CV for GIR-AUC _(0-24h)_ was 20 % for IDeg and 82 % for IGlar (Fig. 3); this difference is probably related to their distinct mechanism of protraction; the IDeg multihexamers at the injection site dissociate slowly to release monomers while IGlar micro precipitates formed at the injection site must re-dissolve before absorption, a process inherently variable.

The clinical implications of the aforementioned pharmacodynamic and PK properties of IDeg at steady-state are once-daily dosing resulting in a low peak: nadir ratio and consequent less variability of action and plasmatic concentrations less critically dependent upon the time of injections, permitting flexibility in dose timing [14].

### Clinical development program (BEGIN)

The clinical development program of IDeg included 3 therapeutic exploratory trials of short duration (6–16 weeks) and 9 therapeutic confirmatory trials of longer duration (26–52 weeks). The primary objectives of the trials were to confirm the efficacy of IDeg administered once daily in controlling glycemia (change from baseline in HbA1c). Table 1 summarizes the studies conducted in T2D (6 studies; *n* =2733 receiving IDeg and *n* =1343 receiving active comparators) and in T1D (3 studies; *n* =1104 receiving IDeg and *n* =474 receiving active comparators). All confirmatory trials were randomized, controlled, parallel-group, open-label, multicenter, multinational and treat-to-target in which IDeg was compared to an active comparator; 5 of the confirmatory trials (3 in T1D and 2 in T2D) were extended for further periods of 26 or 52 weeks to evaluate long-term safety. One trial in T1D and one trial in T2D included a third treatment arm in which IDeg was administered in the morning and in the evening on alternating days (the Fixed Flexible dose schedule) aiming at evaluating the impact of extreme day-to-day variation in the dosing intervals (from 8–12 h to 36–40 h).

**Table 1:** Overview of the therapeutic confirmatory trials of degludec in patients with Type 1 and Type 2 diabetes mellitus

Trial	Population	Therapy	Treatment arms	Treatment combination	Duration (Weeks)	Treatment at screening
3582	Insulin-treated T2D	Basal-bolus	Degludec OD	+ Aspart	52	Any insulin regimen
			*versus*	± Met	(Extension of 26 wks)	
			Glargine OD	± Pio		
3579	Insulin-naïve	Insulin + OADs	Degludec OD	+ Met	52	Met (mandatory) ± SU, ±a-GI, ± DPP-4i in any combination
	T2D		*versus*	± DPP-4i	(Extension of 52 wks)	
			Glargine OD			
3672	Insulin-naïve	Insulin + OADs	Degludec OD	+ Met	26	Met (mandatory) ± SU, ± α-GI, ± DPP-4i in any combination
	T2D		*versus*	± DPP-4i		
			Glargine OD			
3586	Insulin-naïve	Insulin + OADs	Degludec OD	± Met	26	Monotherapy or combination of SU and Met ± α-GI, or DPP-4i
	T2D		*versus*	± SU		
			Glargine OD	± α-GI		
3580	Insulin-naïve	Insulin + OADs	Degludec OD	+1-2 ADOs: Met, SU, Pio	26	± Met, ± SU, ± pio 1–2 OADs in any combination
	T2D		*versus*			
			Sitagliptin OD			
3668	Insulin-naïve + insulin -treated	Insulin + OADs	Degludec Fixed Flex OD *versus*	± Met	26	OADs only or basal insulin only or basal insulin + OADs (any combination of Met, SU or Pio)
	T2D		Glargine OD and Degludec Fixed Flex OD	± SU		
			*versus*	± Pio		
			Degludec OD			
3583	Insulin- treated	Basal-bolus	Degludec OD	+ Aspart	52	Any basal-bolus regimen
	T1D		*versus*		(Extension of 52 wks)	
			Glargine OD			
3585	Insulin- treated	Basal-bolus	Degludec OD	+ Aspart	26	Any basal-bolus regimen
	T1D		*versus*		(Extension of 26 wks)	
			Detemir OD			
3770	Insulin- treated	Basal-bolus	Degludec Fixed Flex OD	+ Aspart	26	Any basal-bolus regimen
	T1D		*versus*		(Extension of 26 wks)	
			Glargine OD and Degludec Fixed Flex OD versus			
			Degludec OD			

*α-GI* alpha-glucosidase inhibitor, *Dpp-4i* dipeptidyl peptidase-4 inhibitor, *Met* metformin, *OADs* oral antidiabetic drugs, *OD* once daily, *Pio* pioglitazone, *SU* sulphonylurea, *T1D* type 1 diabetes, *T2D* Type 2 diabetes, *Wks* weeks

The inclusion criteria concerning antidiabetic therapy and disease duration (mean of 17.3 years for T1D and 10.5 years for T2D) ensured that all subjects were eligible for intensified treatment. The HbA1c limits were 7.0 or 7.5 to 10 or 11.0 % (mean of 7.8 % for T1D and 8.4 % for T2D) and the upper limits for body mass index (BMI) were 35.0 for T1D and 40.0 kg/m^2^ for T2D (except in Trial 3586, that included Asian subjects, for whom the upper limit was 35.0 kg/m^2^ and Trial 3672, in which the upper limit was 45.0 kg/m^2^). Insulin-naïve patients with T2D started once daily basal insulin at a dose of 10 U/day while in insulin-treated subjects, a unit-to-unit transfer was recommended, with adjustments performed at the investigator’s discretion.

**Fig. 4:**
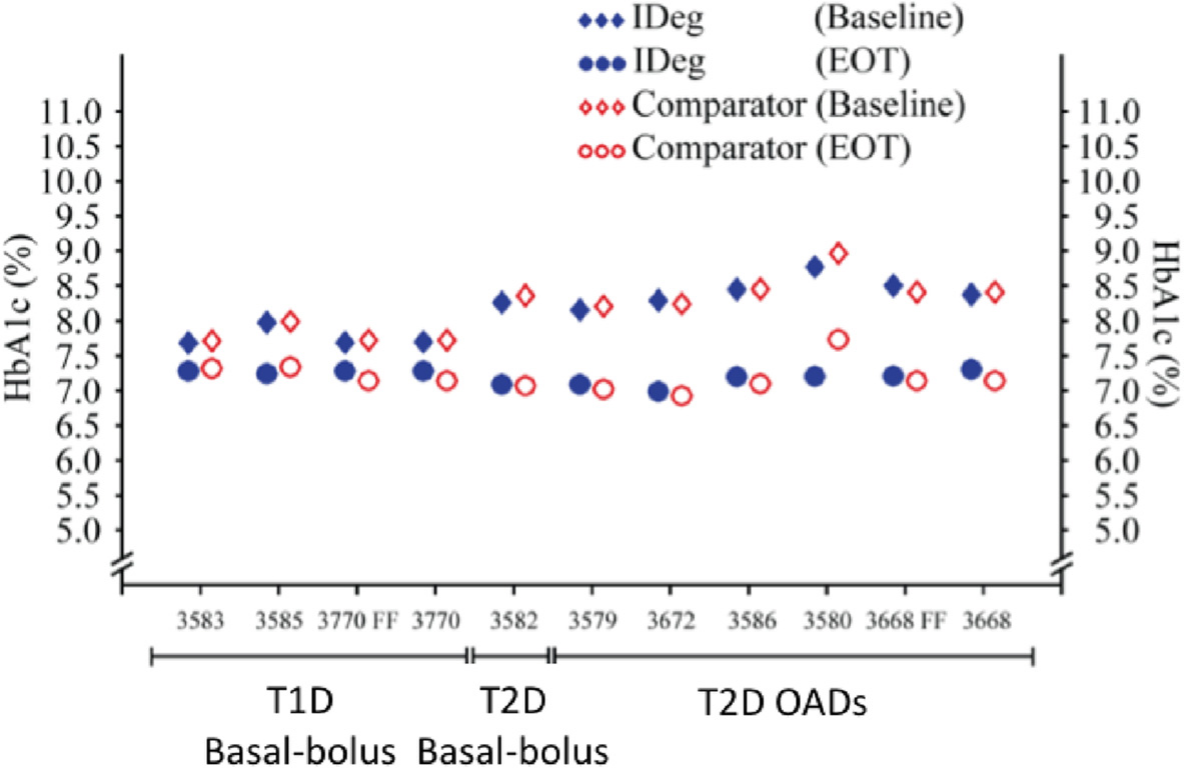
Mean values of HbA1c observed in the 9 therapeutic confirmatory trials at baseline and at end of trial (EOT) for insulin degludec (IDeg) and comparators (insulin glargin, except in trials 3585 [insulin determir] and 3580 [sitagliptin]). FF: Fixed-flexible schedule; OADs: oral antidiabetic drugs; T1D: Type 1 diabetes; T2D: Type 2 diabetes. Adapted from Reference [15]

**Fig. 5:**
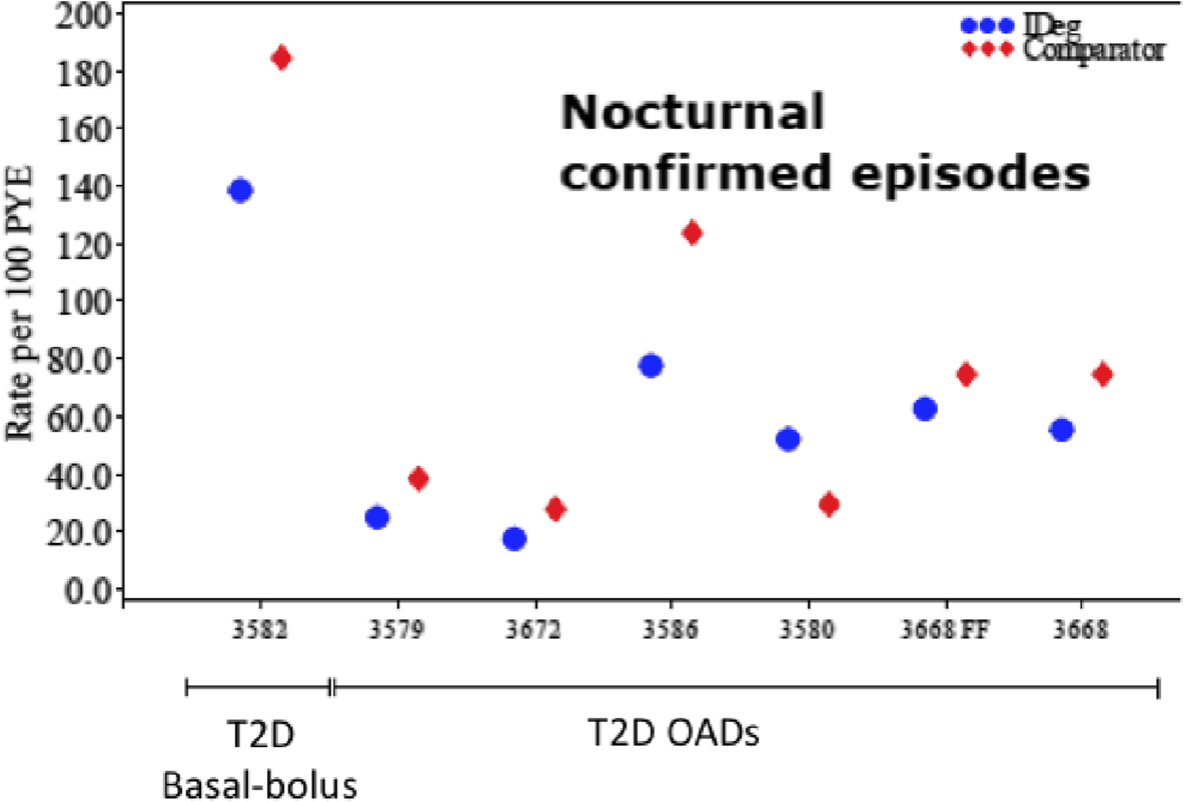
The frequency of confirmed nocturnal hypoglycemia (between 00:01 and 5:59 am) with insulin degludec (IDeg) and comparators (insulin glargin, except in trial 3580 [sitagliptin]) in Type 2 diabetes patients participating in 6 therapeutic confirmatory trials. FF: Fixed-flexible schedule; PYE: patient years of exposure; T1D: Type 1 diabetes; T2D: Type 2 diabetes. Adapted from Reference [15]

The changes in HbA1c observed in the therapeutic confirmatory trials are shown in Fig. 4; efficacy of IDeg was demonstrated in T1D and T2D patients across different age, BMI and ethnic groups, and in combination with different OADs. The Fixed Flexible dose schedule was as effective as IDeg dosed every day in the evening both in T1D and T2D patients. The frequency of confirmed nocturnal hypoglycemia was lower with IDeg than with IGlar in T2D (Fig. 5), while no significant differences were observed in T1D patients, this analysis was probably affected by the low number of severe nocturnal episodes observed along the clinical trials [15].

The risk of hypoglycemia in T2D patients was additionally evaluated in a meta-analysis of 5 confirmatory trials (3582, 3579, 3672, 3586, 3668) that considered the subset of subjects who required high basal insulin doses (>60 U) at the end of the studies (*n* = 795 patients receiving IDeg *versus n* = 374 receiving IGlar for 26 or 52 weeks). Patients in the two treatment arms achieved similar mean HbA1c values (7.2 %), while mean fasting plasma glucose was significantly lower with IDeg (115,4 mg/dL) than with IGlar (119,8 mg/dL, *P* = .04). Lower rates of overall confirmed hypoglycemic episodes and nocturnal confirmed hypoglycemic episodes were observed with IDeg in comparison to IGlar (*P* < .01) [16].

The results of 3 confirmatory trial extensions have been recently published. In Trial 3579 (BEGIN Once Long), 505 of the 773 randomized insulin-naive T2D patients completed 104 weeks of study (52-week main study + 52-week extension period). Mean HbA1c decreased from 8.1 ± 0.8 % and 8.2 ± 0.8 % at baseline to 7.0 ± 0.9 % and 6.9 ± 0.8 % at 104 weeks with IDeg and IGlar, respectively. Overall confirmed hypoglycemia rates were similar between IDeg and IGlar when considering the entire trial period, but nocturnal confirmed hypoglycemia was 43 % lower with IDeg at the end of 104 weeks (rate ratio [RR, expresses the relative chance of hypoglycemia in the group of patients receiving degludec as compared with the group receiving glargine] of 0.57, 95 % confidence interval [CI] 0.40-0.81, *P* = .002) as well as severe hypoglycemia (RR of 0.31, 95 % CI 0.11-0.85, *P* = .023) [17].

In Trial 3583 (BEGIN Basal-Bolus Type 1) 469 of the 629 randomized T1D patients completed 104 weeks of study (52-week main study + 52-week extension period). Mean HbA1c decreased from 7.7 % at baseline in both groups to 7.4 % and 7.5 % at 104 weeks with IDeg and IGlar, respectively; these results were achieved with patients in the IDeg treatment arm receiving 12 % less basal and 9 % less total daily insulin than patients in the IGlar treatment arm (*P* < .01). Overall confirmed hypoglycemia rates were similar between IDeg and IGlar when considering the entire trial period, but nocturnal confirmed hypoglycemia was 25 % lower with IDeg at the end of 104 weeks (RR of 0.75, 95 % CI 0.59-0.95, *P* = .02) [18].

Finally, in Trial 3770 (BEGIN: Flex T1) the Fixed Flexible dose schedule of IDeg was compared to IDeg or to IGlar, both given at the same time daily for 26 weeks to T1D patients. In the 26-week extension period, all IDeg patients were shifted to a free-flexible schedule (Free-Flex; dosing allowed at any time of the day) and compared with patients who continued on IGlar. Mean HbA1c decreased from 7.7 % at baseline in all three groups by −0.40 %, −0.41 % and −0.58 % points with IDeg Fixed Flexible, IDeg and IGlar, respectively at week 26. At the end of the extension period, HbA1c was 7.6 % in the IDeg Free Flex treatment arm and 7.5 % in the IGlar treatment arm, and mean daily basal, bolus and total insulin doses were lower by 4 %, 18 % and 11 %, respectively with IDeg Free Flex. Confirmed and severe hypoglycemia rates were similar among the three groups after 26 weeks, but nocturnal confirmed hypoglycemia was lower with IDeg Fixed Flex than with IGlar (by 40 %) and IDeg (by 37 %). At week 52, nocturnal confirmed hypoglycemia was 25 % lower with IDeg Free-Flex than with IGlar (*P* = .026) [19].

Still regarding IDeg and hypoglycemia, a double-blind randomized crossover study using stepwise hypoglycemic clamp has demonstrated that symptomatic and cognitive responses to induced hypoglycemia, as well as the time required for glycemia recovery are similar between IDeg and IGlar, reassuring the safety profile of IDeg with respect to hypoglycemic awareness [20].

### Use in special populations

The evaluation of the PK profile of IDeg after a single dose in diabetic and non-diabetic subjects presenting normal hepatic function and mild (Child–Pugh grade A), moderate (Child–Pugh grade B) or severe (Child–Pugh grade C) hepatic impairment revealed no differences in the AUC_120-h_ of plasmatic IDeg concentration–time curve, in C_max_ and in the apparent clearance (CL/F) for individuals with impaired *versus* normal hepatic function. Differences in serum albumin concentrations did not interfere with the AUC _120-h_ [21].

PK of a single dose of IDeg was also evaluated in subjects with renal impairment with or without DM (creatinine clearance [CLCR] estimated by the Cockcroft and Gault formula: CLCR 50–80 mL/min [mild], CLCR 30–49 mL/min [moderate], CLCR <30 mL/min [severe] or end-stage renal disease requiring hemodialysis) in comparison to subjects with normal renal function (CLCR >80 mL/min). Again, no differences were observed in the AUC_120-h_, in C_max_ and in CL/F for individuals with impaired *versus* normal renal function [22]. One possible explanation for these findings is that although the liver and kidneys participate in insulin clearance, it also requires internalization of the insulin receptor in the target cells, a process that might be more prevailing in albumin-bound insulins that are not filtered by the kidney as readily‬ as unbound insulins. Thus, hepatic and renal impairment do not significantly interfere with the PK properties of these insulin analogues [23].‬

With respect to the elderly population, there were only minor differences in the PK properties of IDeg between young and elderly subjects [15]. A recent meta-analysis assessed the frequency of hypoglycemia in 917 T1D and T2D patients ≥ 65 years of age who participated in 7 confirmatory trials comparing IDeg and IGlar (3582, 3579, 3672, 3586, 3668, 3583, 3770). In the pooled population of T1D + T2D patients, rates of confirmed nocturnal hypoglycemia were 35 % lower with IDeg than IGlar for the total treatment period (OR 0.65, CI 95 % 0.46-0.93, *P* < .05) [24].

Based on the results from the clinical studies, the type, frequency and severity of adverse reactions observed in elderly patients and in those with renal or hepatic impairment are not different from the general population [15].

## Conclusions

IDeg is an ultra-long insulin analog that exhibits low intra-individual variability and whose efficacy is comparable to IGlar, but which presents as advantages flexibility in dose timing and lower risk of hypoglycemia, benefits that may impact quality of life and adherence to therapy [25]. Thus, this ultra-long insulin analog is a relevant addition to the therapeutic armamentarium for diabetes.

## Abbreviations

AUC: Area under the curve
BMI: Body mass index
CI: Confidence interval
CLCR: Creatinine clearance
Cmax: Maximum plasmatic concentration
CV: Coefficient of variation
DM: Diabetes mellitus
EOT: End of trial
GIR-AUC (0–24 h): Area under the curve for glucose infusion rates from 0–24 h
IGlar: Glargine
OADs: Oral antidiabetic drugs
OR: Odds ratio
PK: Pharmacokinetic
RR: Rate ratio
t1/2: Terminal half-life
T1D: Type 1 diabetes
T2D: Type 2 diabetes
